# Draft Genome Sequence of the First Hypermucoviscous *Klebsiella variicola* Clinical Isolate

**DOI:** 10.1128/genomeA.01352-14

**Published:** 2015-04-09

**Authors:** Ulises Garza-Ramos, Jesus Silva-Sanchez, Humberto Barrios, Nadia Rodriguez-Medina, Jesus Martínez-Barnetche, Veronica Andrade

**Affiliations:** a Instituto Nacional de Salud Pública, Centro de Investigación Sobre Enfermedades Infecciosas, Departamento de Diagnóstico Epidemiológico, Cuernavaca, Morelos, Mexico; b Instituto Nacional de Salud Pública, Centro de Investigación Sobre Enfermedades Infecciosas, Departamento de Inmunología, Cuernavaca, Morelos, Mexico; c Hospital Regional Centenário de la Revolución Mexicana, ISSSTE, Emiliano Zapata, Morelos, Mexico

## Abstract

An antibiotic-susceptible and hypermucoviscous clinical isolate of *Klebsiella variicola* (*K. variicola* 8917) was obtained from the sputum of an adult patient. This work reports the complete draft genome sequence of *K. variicola* 8917 with 103 contigs and an annotation that revealed a 5,686,491-bp circular chromosome containing a total of 5,621 coding DNA sequences, 65 tRNA genes, and an average G+C content of 56.98%.

## GENOME ANNOUNCEMENT

In the last decade, a new hypervirulent (hypermucoviscous) variant of *Klebsiella pneumoniae* has been described (1). Most isolates of hypervirulent *K. pneumoniae* are very susceptible to antimicrobials (except ampicillin). However, a multidrug-resistant and hypervirulent variant of *K. pneumoniae* has also been described as the next “superbug” (2). On the other hand, *Klebsiella variicola* is a Gram-negative rod of the *Enterobacteriaceae* family; it was described as a new bacterial species in 2004 (3). Currently, *K. variicola* is known to be an endophyte of plants (3, 4), a symbiont in insects (5), and a pathogen in humans (3). A susceptible and multiresistant phenotype of *K. variicola* has been identified, corresponding to an extended spectrum β-lactamase (ESBL)–producing *K. variicola*, encoding the SHV-type and CTX-M-15 genes (6, 7).

It is difficult to distinguish *K. variicola* from *K. pneumoniae* biochemically as bacterial species. Therefore, it is necessary to use molecular tools such as the *rpoB* analysis. Accordingly, our teamwork developed a multiplex PCR assay for the proper differentiation of these sister bacteria (7). Using this molecular tool, a screening for antibiotic-susceptible and multiresistant *K. pneumoniae* clinical isolates was carried out in several Mexican hospitals (7). As a result, the susceptible *K. variicola* clinical isolate 8917 was identified. This isolate was obtained from the sputum of a 76-year-old man at the Hospital Regional Centenário de la Revolución Mexicana in Morelos, Mexico, in 2011. This isolate was initially identified as a susceptible (except to ampicillin) *K. pneumoniae* isolate using a MicrosScan Walkaway system (Dade Behring, West Sacramento, CA, USA). Subsequently, it was identified as *K. variicola* using the M-PCR-1; this was confirmed by the phylogeny analysis of the *rpoB* gene (7). The hypermucoviscous phenotype of *Klebsiella variicola* isolate 8917 was determined using the semiquantitative string test (8) and then was considered for whole-genome sequencing.

A total genomic sample of *K. variicola* isolate 8917 was extracted and purified using the DNeasy kit (Qiagen, Germany). The whole-genome sequence was generated using pyrosequencing on the 454 Roche FLX Titanium platform. The sequence data totaled 250,217 reads, with a range in length of 30 to 953 bp. Reads longer than 500 bp were used for *de novo* assembly with the CLC Genomics Workbench version 4.0 (CLC bio). In total, 103 contigs with an *N*_50_ of 257,189 bp were obtained. The estimated genome size was 5,686,491 bp with a 20× coverage, and 99.93% of the bp were above Q40. Gene prediction and annotation were carried out using the bioinformatic MicroScope platform (9). A total of 5,621 coding DNA sequences and 65 tRNA genes were determined. The BLAST searching analysis of the *magA*, *rmpA*, and *rmpA2* genes described in hypervirulent *K. pneumoniae* turned out to be negative on the hypermucoviscous *K. variicola* 8917 genome. However, the following virulence-associated determinants were positive with different amino acid identities: *uge* (99.10%), *ureA* (100%), *wabG* (99.47%), *iroN* (64.9%), *iutA* (72.9%), *kfuABC* (>98.6%), *mceG* (53.1%), *mrkABCDFHIJ* (>86.8%), and *entB* (99.6) and a nucleotide identity of 100% with wzc-932 (serotype). Further analyses are required to identify the genes involved in the hypermucoviscous phenotype on *K. variicola* clinical isolate 8917.

### Nucleotide sequence accession number.

The annotated genome sequence is available at the European Nucleotide Archive under the accession number CEGG01000001.
